# Complete mitogenome of two dolphinfishes (*Coryphaena hippurus* and *Coryphaena equiselis*) from South China sea

**DOI:** 10.1080/23802359.2018.1516120

**Published:** 2018-09-22

**Authors:** Lei Xu, Xuehui Wang, Hong Li, Feiyan Du

**Affiliations:** a South China Sea Fisheries Research Institute, Chinese Academy of Fishery Sciences, Guangzhou, China;; b Guangdong Provincial Key Laboratory of Fishery Ecology and Environment, Guangzhou, China

**Keywords:** Mitochondrial genome, Coryphaena, South China Sea

## Abstract

The Coryphaenidae family comprises of single genus, *Coryphaena*, which includes two species: *Coryphaena hippurus* and *Coryphaena equiselis*. In this study, we described the complete mitochondrial genome of the two species in *Coryphaena*. The assembled mitogenome of *C*. *hippurus* and *C*. *equiselis* consists of 16731 bp and 16690 bp, respectively. Two mitogenomes contain the typical gene complement including 13 protein-coding genes, 22 transfer RNAs, 2 ribosomal RNA genes and a non-coding D-loop. The longest protein-coding genes of these species was *ND5*, whereas the shortest *ATP8*. The length of D-loop is 1168 bp (*C. hippurus*) and 1206 bp (*C. equiselis*).

The Coryphaenidae family is comprised of single genus, *Coryphaena*, which only includes two species: *Coryphaena hippurus* and *Coryphaena equiselis* (Collette 1999). The dolphinfish is widely distributed in tropical and subtropical waters throughout the Atlantic, Indian and Pacific Oceans including the Mediterranean Sea (Gibbs and Collette 1959). Here, we sequenced and annotate mitogenome of two species, *C. hippurus* and *C. equiselis* in *Coryphaena* from South China Sea to provide molecular information for genetically understanding of dolphin fishes. The specimens of *C. hippurus* and *C. equiselis* were collected from two locations in the South China Sea (21°25′N, 117°02′E and 20°28′N, 120°03′E) in September 2017. Whole genomic DNA was extracted from muscle tissue of one specimen of each species using TIANamp Marine Animals DNA Kit (TIANGEN, China). The concentration for use as a PCR template was adjusted to an A_260_ of about 0.05 to 0.2. The collected specimens and extracted DNA were stored in Guangdong Provincial Key Laboratory of Fishery Ecology and Environment. The complete mitochondrial genomes were sequenced using PCR primers designed from highly conserved regions of transfer RNA (tRNA) sequences of congeneric species (Diaz-Jaimes et al. 2015) with additional specific primers designed as required from sequences already obtained. Long-PCR amplifications were performed by thermo-cycling using five pairs of primers and PCR amplicons were subjected to build up genomic library and pair-end sequencing by MiSeq. The COI sequence was used as reference seeds for iterative assembly by MITObim v.1.8 (Hahn et al. 2013). SeqMan v.7.1.0 was used for the mitogenome assembly and annotation (Swindell and Plasterer 1997). Transfer RNA genes were predicted using online software tRNAScan-SE 1.21 (Lowe and Eddy 1997). All the Protein coding gene (PCGs) are aligned independently, then concatenated to be applied for phylogenetic reconstruction with other Carangiformes in MrBayes v 3.12 (Ronquist and Huelsenbeck 2003) using relaxed clock model.

Complete mitogenomes of *C. hippurus* and *C. equiselis* consist of 16731 bp (GeneBank: MH576915) and 16690 bp (GeneBank: MH576916), respectively. These mitogenomes contain the typical gene complement including 13 protein-coding gene, 22 transfer RNA genes and 2 rRNA genes (12S rRNA and 16S rRNA) and one A + T-rich region which could also be termed as control region. The overall base composition of the whole mitochondrial genome of *C. hippurus* was A (26.69%), T (27.20%), G (15.61%) and C (23.88%) with an AT bias of 53.89%. The overall base composition of *C. equiselis* was A (27.11%), T (28.55%), G (16.71%), and C (24.32%) with an AT bias of 55.66%. In the two species, the ATG initiation codon are used in all protein-coding genes except *COX1* (GTG) and *ATP8* (ATT), the stop codons of all the 13 protein-coding genes were complete. Meanwhile, the longest protein-coding genes of these species was *ND5* (1818 bp in *C. hippurus*; 1839 in *C. equiselis*), whereas the shortest *ATP8* (165 bp in *C. hippurus*; 168 in *C. equiselis*). *lrRNA* and *srRNA* genes are 1719 bp and 946 bp in length in *C. hippurus*, 1721 bp and 946 bp in length in *C. equiselis*, and the length of D-loop is 1168 bp (*C. hippurus*) and 1206 bp (*C. equiselis*). All the 22 typical tRNAs possess a complete clover leaf secondary structure, ranging from 66 bp to 75 bp. The Bayesian inference phylogenetic tree showed that the two species firstly grouped together (Figure 1). We have the confidence to construct phylogenetic trees, based on the complete the mitochondrial genomes, but the evolution history of dolphinfishes still needs future research to be clearly resolved.

**Figure 1.**
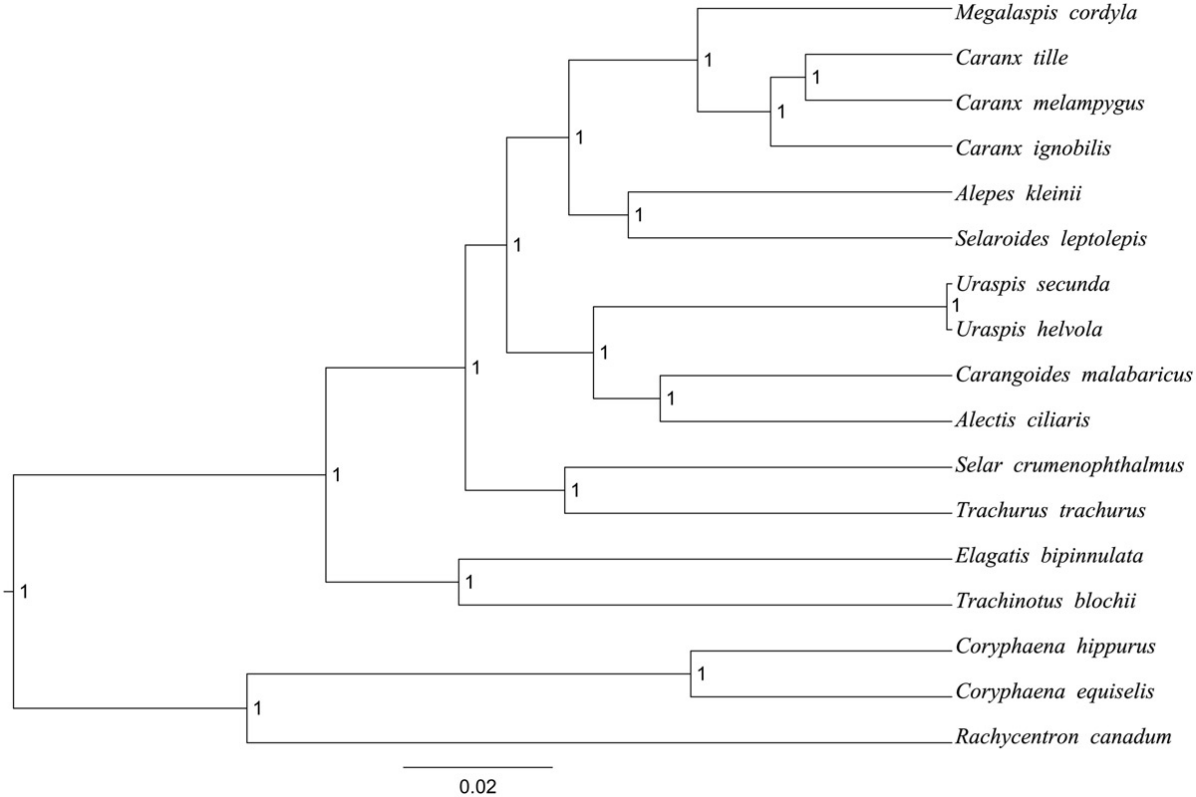
The Bayesian inference phylogenetic tree for Carangiformes based on mitochondrial PCGs and rRNAs concatenated dataset. The gene’s accession numbers for tree construction are listed as follows: *Elagatis bipinnulata* (KT824759), *Uraspis helvola* (KM978993), *Uraspis secunda* (KT819204), *Megalaspis cordyla* (KM522836), *Alepes kleinii* (KF728081), *Carangoides malabaricus* (KJ174514), *Selaroides leptolepis* (KM522839), *Trachurus trachurus* (AB108498), *Trachinotus blochii* (KJ184305), *Caranx ignobilis* (KF649842), *Caranx melampygus* (KF649843), *Caranx tille* (KT805946), *Alectis ciliaris* (KM522837), *Selar crumenophthalmus* (KJ148633), *Rachycentron canadum* (FJ154956).
